# Determinants of stunting among children under 2 years in urban informal settlements in Mumbai, India: evidence from a household census

**DOI:** 10.1186/s41043-020-00222-x

**Published:** 2020-11-27

**Authors:** Sushmita Das, Sheila Chanani, Neena Shah More, David Osrin, Shanti Pantvaidya, Anuja Jayaraman

**Affiliations:** 1 Society for Nutrition, Education and Health Action, Behind Bldg. No. 11, BMC Colony, Shastri Nagar, Santa Cruz (W), Mumbai, 400 054 India; 2 UCL Institute for Global Health, 30 Guilford Street, London, WC1N 1EH UK

**Keywords:** Malnutrition, Stunting, Slum settlements, Mumbai, India

## Abstract

**Background:**

There is limited evidence on the determinants of childhood stunting across urban India or specifically in slum settlements. This study aims to assess the extent of stunting among children under 2 years of age and examine its determinants in informal settlements of Mumbai.

**Methods:**

Data were collected in 2014–2015 in a post intervention census of a cluster randomized controlled trial to improve the health of women and children. Census covered 40 slum settlements of around 600 households each. A total of 3578 children were included in the study. Mixed effects logistic regression models were used to identify factors associated with stunting.

**Results:**

The prevalence of stunting among children aged 0–23 months was 38%. In the adjusted model, higher maternal education (AOR 0.59; 95% CI 0.42, 0.82), birth interval of at least 2 years (AOR 0.71; 95% CI 0.58, 0.87) and intended conception of the child (AOR 0.80; 95% CI 0.64, 0.99) were associated with lower odds of stunting. Maternal exposure to physical violence (AOR 1.83; 95% CI 1.21, 2.77) was associated with higher odds of being stunted. A child aged 18–23 months had 5.04 times greater odds (95% CI 3.91, 6.5) of being stunted than a child less than 6 months of age. Male child had higher odds of being stunted (AOR 1.33; 95% CI 1.14, 1.54).

**Conclusions:**

Our findings support a multidimensional aetiology for stunting. The results of the study emphasize the importance of women’s status and decision-making power in urban India, along with access to and uptake of family planning and services to provide support for survivors of domestic violence. Ultimately, a multilateral effort is needed to ensure the success of nutrition-specific interventions by focusing on the underlying health and social status of women living in urban slums.

**Trial registration:**

ISRCTN Register: ISRCTN56183183, and Clinical Trials Registry of India: CTRI/2012/09/003004

## Background

The last two decades have witnessed encouraging global trends in the reduction of childhood stunting. Between 2000 and 2016, stunting prevalence among children under five declined from 33 to 23% and the global number of stunted children declined from 198.4 million to 154.8 million [1]. India, home to one third of stunted children [2], has shown similar downward trends. The recent National Family Health Survey (NFHS 2015–2016) [3] indicated that child stunting had fallen from 48 to 38% over one decade—nearly doubling the rate of stunting reduction in previous decades [4]. In spite of this remarkable progress, measures are still needed to accelerate reductions further to achieve the global nutrition targets adopted by the World Health Assembly for 2025 [5].

In Mumbai, India’s largest metropolis, 42% of the population live in slums [6]. It is estimated that 26% of children in Mumbai are stunted, but data have not been disaggregated for slum areas [7]. Malnutrition rates among children in slums are generally higher than in non-slum areas. Studies conducted in Mumbai’s slum settlements have found prevalence of stunting of 34 to 47% among children under five [8–10].

Slum populations rank among the poorest and most underserved communities in India. The census of India defines a slum as “areas unfit for human habitation due to dilapidation, overcrowding, faulty building design, narrow or faulty arrangements of streets, lack of ventilation, light or sanitation facilities, or any combination of these factors detrimental to safety and health” [6]. Inadequate access to safe drinking water and sanitation services puts children at increased risk of illness, malnutrition and death. Evidence suggests that the risks exceed those prevalent in rural areas [11].

Stunting results from chronic suboptimal nutrient intake, thereby restricting a child’s growth, and is defined as height-for-age less than two standard deviations below the WHO Child Growth Standards median [12]. Stunting has severe short- and long-term health and functional consequences, including poor cognition and educational performance, low adult wages and lost productivity [13]. The harms are largely irreversible and have negative consequences through to adulthood [14]. A framework developed by UNICEF recognizes that the determinants of stunting are multidimensional due to an interaction of household, environmental, socio-economic and cultural influences [15]. While the factors that contribute to childhood stunting are complex, a window of opportunity exists within the first 1000 days of a child’s life when linear growth is most sensitive to environmentally modifiable factors, such as feeding, morbidity treatment and psychosocial care. Growth trajectories are set early in life and 70% of stunting reportedly occurs during this period [16].

There is limited evidence on the determinants of childhood stunting across urban India or specifically in slum settlements. A review of the current literature available for urban India suggests that determinants of malnutrition among children include poor maternal health (including body mass index and nutrition), low parental educational status, compromised household sanitation and hygiene, respiratory and gastrointestinal illnesses, underutilization of health care services, low birth weight and poor child feeding practices [17–19].

Our study drew on a census of families in 40 slum settlements of Mumbai covering a population of 120,000. Its objectives are (1) to document the extent of stunting and (2) to examine the determinants of early childhood stunting among children under 2 years.

## Methods

### Study design

Data for the study originated from a trial to evaluate the impact of community resource centres on maternal and child health and nutrition outcomes. Data were collected from February 2014 to September 2015, in a census after the trial intervention covering all households with married women aged 15–49 years. We used these cross-sectional data in a secondary analysis to understand the extent and determinants of stunting in children under 2 years of age in the study population. The original trial tested a model that included service provision, outreach and community mobilization activities, cascaded out through the community resource centres. The resource centres targeted women of reproductive age and children under 5 years of age to participate in activities and access health and nutrition services. The results of the trial are presented elsewhere [20].

### Setting

The commercial capital of Maharashtra state, Mumbai, is India’s most populous city. The existing infrastructure is overburdened and nearly half of the population lives in slum settlements. The Municipal Corporation of Greater Mumbai is the primary local body responsible for civic administration across 24 municipal wards in three zones: city, central and western. The sampling frame of this study included two of Mumbai’s most vulnerable municipal wards (M East and L) in the central zone, with the lowest Human Development Indices. The low indices of these wards could be attributed to large migrant populations, low and insecure levels of livelihood activity, large-scale unauthorized housing and poor education and health facilities [21]. The study areas were selected after a systematic vulnerability assessment in both wards using a vulnerability score card [22]. Each area consisted of ~ 600 households; some areas included entire slum clusters, while others were sections of larger geographical settlements. Most of the 40 areas involved in the study were situated on or beside hazardous locations like railway lines, garbage dumps, creeks and open sewers or drains. The primary health services in these areas were provided by municipal health centres and included outreach services for antenatal care and immunization for children. The population also had access to government child care centres, providing health, education and nutrition services, growth monitoring, supplementary nutrition, preschool education, immunization, nutrition, health education and referrals.

All the households in the study population held a ration card, an official document provided to households that are eligible to purchase subsidized food grains from the public distribution system. The majority of participants held orange ration cards given to households with annual income between INR 15,000 and 100,000 (US$ 204–1360).

### Sample

**Fig. 1:**
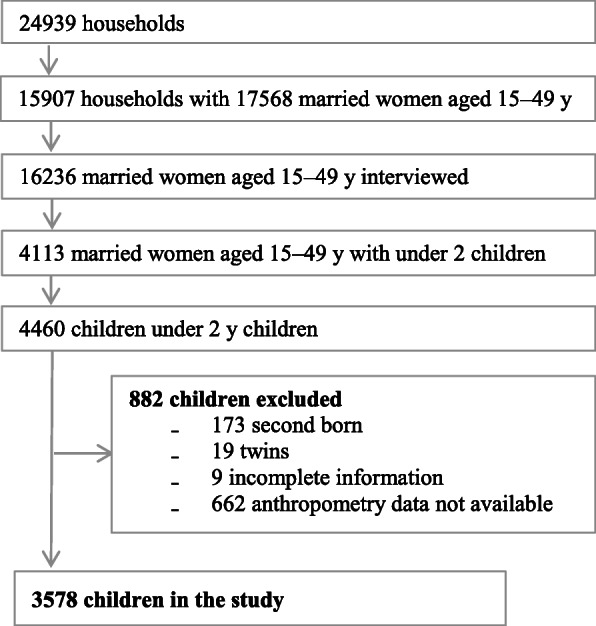
Study profile of children under 2 years in 40 informal settlements of Mumbai, 2014–2015

The trial covered 24,939 households of which 15,907 households had married women in the reproductive age group. We could interview 16,236 out of 17,568 eligible women in 15,907 households. 4113 (25%) of all women interviewed had a total of 4460 children under 2 years of age. Of all (4460) children under two, 173 had a younger sibling. In the case of two siblings under 2 years of age, we excluded the older sibling from the analysis as the socio-demographic profile of the mother remained same and recall period was longer as compared to the younger child. Twins (19) were also excluded from the analysis as their nutritional status differs from that of singletons [23]. Complete information was not available for 9 children and anthropometric data were missing for 662 children. The final sample included 3578 children under the age of two (Fig. 1).

### Data collection

Two teams of six investigators and one supervisor collected the data for the census. Each team was responsible for data collection in 20 study areas. The primary respondents were mothers of children 0–2 years of age. Investigators visited homes up to three times each to set up interviews with the mothers. The questionnaire was administered in Hindi to eligible women, after which weight and length of all children were measured.

#### Anthropometric assessment

Anthropometric measurements were used in assessment of nutritional status of all children aged 0–23 months. Weight was measured on Seca 385 electronic scales to a precision of 10 g. Length of children was measured with Harlow rollameters to a precision of 1 mm. All children were measured twice and the mean value was used in the analysis. To minimize intra- and inter-observer errors, relative technical error of measurement (% TEM) was evaluated on two occasions during the data collection period, with representative values of 0.38% and 0.50% for height.

#### Other variables

At the household level, information was collected on home ownership, housing construction, drinking water source and toilet facility. Parental age, educational attainment and current employment status were also part of the questionnaire. Information on maternal care included intended pregnancy, antenatal care and place of delivery. The module on the child included information on age, sex, birth interval and immunization. Information on Infant and Young Child Feeding (IYCF) was collected according to WHO guidelines [24]. Women also answered an additional set of questions about intimate partner violence in the 2 years preceding the census.

Data were collected on smartphones with an open-source tool from Open Data Kit (Seattle, WA, USA) running in Google Android (versions 3.0–4.4). The system included automatic skips and validation constraints. Five percent of interviews selected at random were observed by a supervisor. Data were checked after download for errors in key fields by a data manager.

### Statistical analysis

#### Outcome variable

The outcome of interest was stunting in children aged 0–23 months, identified by length/height measurement. Children with height-for-age *z* scores below minus two standard deviations (HAZ < − 2SD) from the median of the WHO growth standards reference population were considered stunted. Children with height-for-age *z* scores below − 6 or above 6 were excluded from the analysis [25].

#### Independent variables

The primary analysis developed a series of mixed effects logistic regression models, including the variable describing stunting and a random effect for cluster. To explore the determinants of stunting, independent variables were chosen purposefully using the WHO framework for household causes of stunted growth and development [26]. This framework groups causes in four broad categories: household and family factors (maternal factors and home environment), inadequate complementary feeding (poor quality foods, inadequate practices and food and water safety), breastfeeding (inadequate practices) and infection (clinical and subclinical infection) [27].

Based on the available data from the census, regression analysis for household and family factors included drinking water source, toilet facility and socio-economic status, intended pregnancy, antenatal care, institutional delivery and birth interval. A variable describing mothers’ experience of physical violence was also included. The analysis also included typical demographic covariates of paternal education, maternal education and occupational status and religion. Demographic characteristics of the child included were age and gender. The components of inadequate complementary feeding and breastfeeding included a proxy variable for “age-appropriate feeding”. This variable was constructed by combining IYCF indicators on exclusive breastfeeding for children under 6 months with minimum acceptable diet for children aged 6–23 months. A child was considered to have age-appropriate feeding if she was under 6 months and exclusively breastfed or aged 6–23 months and consumed a minimally acceptable diet [28].

We described the characteristics of households, mothers, fathers and children with frequencies and proportions. We used means and standard deviations (SDs) to describe continuous variables. We developed an index of household wealth from standardized weights for the first component of a principal components analysis of data on household assets and split the resultant factor scores into quartiles [29]. We generated standard deviation (*z*) scores for height for age using the zscore06 module in Stata/IC 13.1 (StataCorp, College Station, TX). We plotted *z* scores against child age and fitted quadratic regression lines with 95% confidence intervals (CI).

### Ethical considerations

We identified no risks of harm to women and children participating in the study. However, if investigators identified children as malnourished or women were concerned about access to health facilities for maternal or child care or were survivors of domestic violence, they were supported through the system by the SNEHA health workers who visited the households regularly. Participants were asked for written consent to interview after an explanation of the purpose, benefits and risks of the study. The right of participants to withdraw from the interview or not to participate was respected. Anonymity of informants was ensured and they were assured of the confidentiality of data.

## Results

Table 1 summarizes the background characteristics of the children. More than half of families said that they owned their homes, most of which were of robust construction. More than three fourths of families received drinking water from public taps or community tap stands and over 80% of households used public toilets. Most families were of Muslim faith. More than half of mothers had secondary education, as did nearly two thirds of fathers. Most fathers were employed, with 59% engaged in a skilled job. Few mothers were engaged in paid work.

**Table 1:** Socio-economic and demographic characteristics, for children under 2 years

Households	3578				
	*n*	%		*n*	%
**Home ownership**			**Socio-economic status**		
Own home	1877	52	Poorest	911	25
Rented home	1701	48	Quartile 2	881	25
**Housing construction**			Quartile 3	950	27
Robust (pucca)	2623	73	Least poor	836	23
Temporary (kaccha)	955	27	**Religion**		
**Drinking water source**			Muslim	2997	84
Public/community tapstand	2777	78	Hindu	575	16
Water tap at home	801	22	Other	6	<1
**Toilet facility**					
Public/shared	2974	83			
Private	604	17			
**Mother**			**Father**		
**Age (years)**			**Age (years)**		
15–19	68	2	≤ 24	237	6
20–24	1194	33	25–29	1142	32
25–29	1360	38	30–34	1094	31
30+	956	27	35+	1047	29
**Education**			Missing	58	2
No formal schooling	977	27	**Education**		
Primary	171	5	No formal schooling	761	21
Secondary	2101	59	Primary	167	5
Higher	329	9	Secondary	2244	63
**Occupation**			Higher	345	9
Employed	149	4	Missing	61	2
Not employed	3429	96	**Occupation**		
**Parity**			Skilled job	2128	59
1 or 2 children	1930	54	Unskilled job	1333	37
3 or more children	1648	46	Not employed	59	2
**Marital status**			Missing	58	2
Married/cohabitating	3520	98			
Widowed/divorced/separated	58	2			

Antenatal care and institutional delivery by mothers of children under two were high: 89% had accessed antenatal care at least four times during pregnancy and 88% had an institutional delivery. Eighteen percent of children under 2 years were born within 2 years of another sibling, shorter than WHO recommendations for spacing [30]. The proportions of boys and girls were similar (Table 2).

**Table 2:** Child characteristics, for children under 2 years

Child	*n*	%
**Age (months)**		
0–5	861	24
6–11	987	28
12–17	943	26
18–23	787	22
**Sex**		
Female	1795	50
Male	1783	50
**Preceding birth interval with elder sibling**		
< 24 months	644	18
≥ 24 months	1986	56
First child	948	26
**Mother had antenatal care**		
4 or more visits	3196	89
No or less than 4 visits	382	11
**Place of birth**		
Hospital	3140	88
Home	438	12

Table 3 provides detailed information on Infant and Young Child Feeding practices. Less than half of infants were reported as having been breastfed within 1 h of birth, and almost two thirds of infants under 6 months were exclusively breastfed. Continued breastfeeding at 1 year was high (81%). Fifty-seven percent of infants aged 6–8 months had been introduced to solids and semi-solids. For children aged 6–23 months, consumption of iron-rich foods was low at 17%. Composite indicators were similarly poor; only 19% met dietary diversity standards and 12% had a minimally acceptable diet. The proportion of children receiving a minimum number of meals (66%) was comparatively better. Age-appropriate feeding, a combined IYCF indicator to measure diet of all children under 2 years, was low at 24%.

**Table 3:** Infant and Young Child Feeding indicators, overall and by age group, for children under 2 years

	Children	*n*	%
**Early initiation of breastfeeding (0–23 months)**	3578	1614	45
**Exclusive breastfeeding under 6 months (0–5 months)**	861	531	62
**Continued breastfeeding at 1 year (12–15 months)**	587	475	81
**Introduction of solids or semi-solids (6–8 months)**	441	251	57
**Minimum dietary diversity (6–23 months)**			
All	2684	514	19
6–11 months	954	81	8
12–23 months	1730	433	25
**Minimum meal frequency (6–23 months)**			
All	2684	1779	66
6–11 months	954	546	57
12–23 months	1730	1233	71
**Minimum acceptable diet (6–23 month)**			
All	2684	321	12
6–11 months	954	61	6
12–23 months	1730	260	15
**Consumption of iron-rich foods (6–23 month)**			
All	2684	460	17
6–11 months	954	155	16
12–23 months	1730	305	18
**Age appropriate feeding (All)**	3545	852	24

Information on IYCF missing for 33 children. Age appropriate feeding defined as exclusive breastfeeding for children under 6 months and minimum acceptable diet for 6–23 months

**Fig. 2:**
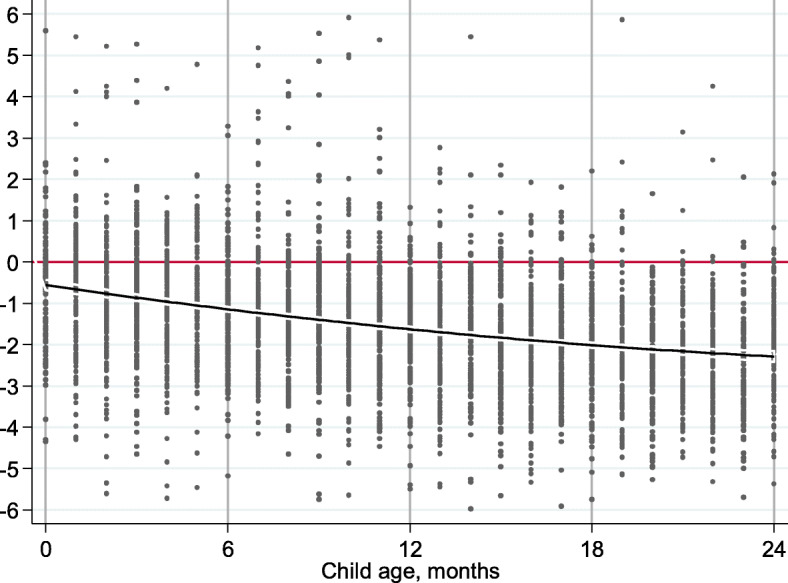
Standard deviation (*z*) scores for stunting by age, showing quadratic line of fit with 95% confidence intervals, for children under 2 years

Table 4 summarizes the anthropometric findings. Children’s lengths or heights were expressed as height-for-age *z* scores (HAZ), which represent an individual child’s stature relative to the WHO reference population median. A HAZ score of − 1 implies that the child is one standard deviation shorter than the median. Conventionally, children whose stature is shorter than two standard deviations below the median (HAZ below − 2) are categorized as stunted. We found that 38% of children were stunted and 14% were severely stunted (HAZ below − 3). Mean height for age was − 1.53 *z* scores, but there was a difference between girls (− 1.46 *z* scores) and boys (− 1.59 *z* scores). The proportion of stunting increased from 21% at 0–5 months to 57% at 18–23 months. This variation in the prevalence is explained in Fig. 2 which depicts length/height for age (HAZ) scores for each child by age. It illustrates the steep fall in length/height for age from birth to around 2 years of age.

**Table 4:** Standard deviation (*z*) scores for anthropometric indicators, overall and by sex and age group, for children under 2 years

Height for age	Children, *N*	*z* score, Mean	SD	*z* score < 2, *n*	%	*z* score < 3, *n*	%
**All**	3578	− 1.53	1.50	1345	37.5	508	14.2
**Boys**	1783	− 1.59	1.56	708	39.7	303	16.9
**Girls**	1795	− 1.46	1.42	637	35.4	205	11.4
0–5 months	861	− 0.85	1.50	182	21.0	47	5.4
6–11 months	987	− 1.30	1.49	298	30.1	91	9.2
12–17 months	943	− 1.82	1.33	419	44.4	157	16.6
18–23 months	787	− 2.21	1.33	446	56.6	212	26.9

Given that growth faltering is most critical in infants and children under 2 years of age, determinants were analysed for this age group (Table 5). The results show that prevalence of stunting was higher in households with shared toilet facility and poor wealth status. Stunting increased with low level of parental education, maternal abuse, narrow birth interval, unintended pregnancy, inadequate antenatal visits and home births. Boys had higher levels of stunting as compared to girls. Stunting increased with age of the child and inadequate age-appropriate feeding.

**Table 5:** Prevalence of stunting by household, parental and child characteristics, for children under 2 years

	Stunting		
	*n*	%	*N*
**Drinking water source**			
Public/community tapstand	1063	38	2777
Water tap at home	282	35	801
**Toilet facility**			
Public/shared	1152	39	2974
Private	193	32	604
**Socio-economic status**			
Poorest	374	41	911
Quartile 2	355	40	881
Quartile 3	339	36	950
Least poor	277	33	836
**Religion**			
Muslim	1116	37	2997
Hindu	228	40	575
Other	1	-	6
**Father’s education**			
No formal schooling	306	40	761
Primary	71	43	167
Secondary	839	37	2244
Higher	103	30	345
**Mother’s education**			
No formal schooling	420	43	977
Primary	69	40	171
Secondary	766	36	2101
Higher	90	27	329
**Mother’s occupation**			
Working	64	43	149
Not working	1281	37	3429
**Mother experienced physical violence**			
Yes	67	54	124
No	1254	37	3393
**Birth interval**			
< 24 months	291	45	644
≥ 24 months	708	36	1986
First child	346	37	948
**Intended pregnancy**			
Yes	1004	36	2832
No	207	42	499
**Antenatal care**			
4 or more ANC visits	1171	37	3196
None or less than 4 visits	174	46	382
**Place of delivery**			
Hospital	1159	37	3140
Home	186	42	438
**Gender**			
Girl	637	35	1795
Boy	708	40	1783
**Child age (months)**			
0–5	182	21	861
6–11	298	30	987
12–17	419	44	943
18–23	446	57	787
**Age appropriate feeding**			
No	1071	40	2693
Yes	262	31	852

Table 6 shows unadjusted and adjusted odds ratios for the association of stunting with parental and household characteristics. In the fully adjusted model, a child was less likely to be stunted if her mother had received higher education above grade 12 (AOR 0.59; 95% CI 0.42, 0.82). For maternal characteristics related to pregnancy, both appropriate spacing of at least 2 years (AOR 0.71; 95% CI 0.58, 0.87), compared with less than 2 years, and intended conception of the child (AOR 0.80; 95% CI 0.64, 0.99) were associated with lower odds of stunting. If a mother had experienced physical violence in the last 2 years, her child had higher odds (AOR 1.83; 95% CI 1.21, 2.77) of being stunted. Males had higher odds of being stunted (AOR 1.33; 95% CI 1.14, 1.54). A child aged 18–23 months had 5.04 times greater odds (95% CI 3.91, 6.5) of being stunted than a child less than 6 months of age.

**Table 6:** Association between household, parental and child level characteristics and stunting in children under 2 years

	Unadjusted OR	I: Home AOR (95% CI)^a^	II: Home and maternal AOR (95% CI)^b^	III: Home, maternal and care practices AOR (95% CI)^c^	IV: Home, maternal, care and feeding practices AOR (95% CI)^d^	V: Full model AOR (95% CI)^e^
***N***		**3517**	**3270**	**3270**	**3237**	**3234**
*β*_o_ (se)		0.76 (.08)	1.05 (0.15)	1.25 (0.23)	1.35 (0.25)	0.47 (0.11)
Water tap at home	0.99 (0.82, 1.21)	1.21 (0.97, 1.51)	1.21 (0.97, 1.52)	1.23 (0.98, 1.54)	1.23 (0.98, 1.55)	1.12 (0.88, 1.42)
Private toilet	0.78 (0.63, 0.96)	0.81 (0.64, 1.03)	0.82 (0.65, 1.05)	0.82 (0.64, 1.05)	0.82 (0.64, 1.05)	0.84 (0.65, 1.09)
Socio-economic Status						
Poorest	1.00	1.00	1.00	1.00	1.00	1.00
Quartile 2	0.93 (0.76, 1.12)	0.96 (0.79, 1.17)	1.00 (0.81, 1.23)	1.02 (0.83, 1.26)	1.04 (0.84, 1.28)	0.97 (0.78, 1.21)
Quartile 3	0.78 (0.64, 0.95)	0.83 (0.68, 1.02)	0.86 (0.69, 1.06)	0.88 (0.71, 1.09)	0.87 (0.70, 1.09)	0.83 (0.66, 1.04)
Least poor	0.73 (0.59, 0.90)	0.81 (0.64, 1.02)	0.85 (0.67, 1.08)	0.87 (0.68, 1.11)	0.90 (0.70, 1.14)	0.87 (0.68, 1.13)
Maternal education						
No formal schooling	1.00	1.00	1.00	1.00	1.00	1.00
Primary	0.92 (0.66, 1.29)	0.92 (0.66, 1.30)	0.95 (0.66, 1.36)	0.97 (0.68, 1.39)	1 (0.69, 1.43)	1.02 (0.7, 1.49)
Secondary	0.76 (0.65, 0.90)	0.79 (0.67, 0.93)	0.8 (0.67, 0.95)	0.81 (0.68, 0.97)	0.83 (0.70, 0.99)	0.84 (0.7, 1.01)
Higher	0.52 (0.40, 0.70)	0.59 (0.44, 0.78)	0.56 (0.41, 0.77)	0.57 (0.42, 0.78)	0.58 (0.42, 0.79)	0.59 (0.42, 0.82)
Experienced physical violence	2.08 (1.44, 3.01)	1.98 (1.36, 2.86)	1.92 (1.3, 2.85)	1.90 (1.28, 2.81)	1.86 (1.25, 2.77)	1.83 (1.21, 2.77)
Birth interval						
< 24 months	1.00		1.00	1.00	1.00	1.00
≥ 24 months	0.67 (0.56, 0.80)		0.69 (0.57, 0.84)	0.7 (0.57, 0.85)	0.68 (0.56, 0.83)	0.71 (0.58, 0.87)
First child	0.70 (0.57, 0.87)		0.78 (0.62, 0.98)	0.79 (0.62, 1.00)	0.77 (0.61, 0.97)	0.79 (0.62, 1.01)
Intended pregnancy	0.78 (0.64, 0.95)		0.85 (0.69, 1.05)	0.86 (0.70, 1.06)	0.83 (0.68, 1.03)	0.80 (0.64, 0.99)
4 or more antenatal care visits	0.70 (0.56, 0.87)			0.77 (0.60, 1.00)	0.78 (0.6, 1.01)	0.77 (0.59, 1.01)
Institutional delivery	0.80 (0.65, 0.99)			1.02 (0.80, 1.29)	1.05 (0.82, 1.34)	1.08 (0.84, 1.39)
Age appropriate feeding	0.66 (0.56, 0.78)				0.67 (0.56, 0.80)	1.03 (0.83, 1.27)
Mother working	1.24 (0.89, 1.74)					1.17 (0.8, 1.72)
Gender: Male	1.21 (1.05, 1.39)					1.33 (1.14, 1.54)
Child age (months)						
0–5 months	1					1.00
6–11 months	1.62 (1.31, 2.02)					1.59 (1.23, 2.05)
12–17 months	3.03 (2.45, 3.74)					3.05 (2.39, 3.89)
18–23 months	5.06 (4.06, 6.30)					5.04 (3.91, 6.5)
Religion						
Muslim	1					1.00
Hindu	1.18 (0.96, 1.46)					1.13 (0.89, 1.43)
Other	0.35 (0.04, 3.10)					0.34 (0.03, 3.33)
Father’s education						
No formal schooling	1					1.00
Primary	1.14 (0.81, 1.61)					1.24 (0.84, 1.83)
Secondary	0.91 (0.77, 1.08)					1.07 (0.88, 1.31)
Higher	0.69 (0.52, 0.91)					0.86 (0.62, 1.20)
SD (se)		0.29 (0.05)	0.26 (0.05)	0.27 (0.05)	0.27 (0.05)	0.28 (0.06)
ICC (se)		0.02 (0.01)	0.02 (0.01)	0.02 (0.01)	0.02 (0.01)	0.02 (0.01)

^a^Model I included variables on water tap at home, private toilet, socio-economic status, mother experiencing physical violence and maternal education

^b^Model II included variables on water tap at home, private toilet, socio-economic status, mother experiencing physical violence, maternal education, birth interval and intended pregnancy

^c^Model III included variables on water tap at home, private toilet, socio-economic status, mother experiencing physical violence, maternal education, birth interval, intended pregnancy, 4 or more antenatal care visits and institutional delivery

^d^Model IV included variables on water tap at home, private toilet, socio-economic status, mother experiencing physical violence, maternal education, birth interval, intended pregnancy, 4 or more antenatal care visits, institutional delivery and age-appropriate feeding

^e^Model V included variables on water tap at home, private toilet, socio-economic status, mother experiencing physical violence, birth interval, intended pregnancy, 4 or more antenatal care visits, institutional delivery, age-appropriate feeding, parental education, religion, gender and age of the child

## Discussion

Our analysis of 3578 children under 2 years of age confirms that children living in urban slums experience impaired growth. At 38%, the prevalence of stunting was considerably higher than global (23%) [1] and urban estimates for Maharashtra (20%) [17]. This could be attributed to the disadvantaged nature of the population. The prevalence of malnutrition in slums is usually higher than the non-slum population [7] and the findings of our study accord with previous studies conducted in slums [9, 31–33]. Health and nutrition of slum children are often compromised and this is further aggravated by lack of basic amenities like adequate housing, safe drinking water and sanitation. A growing body of evidence suggests that water, sanitation and hygiene (WASH) are important determinants of childhood stunting [34, 35]. Though 27% of houses in our study were of insubstantial construction and high proportions did not have access to individual piped drinking water (78%) or private toilets (83%), we did not find a significant association of these factors with stunting. One of the probable explanations could be that they were defined with minimum benchmarks of piped water supply and basic household sanitation.

Stunting often begins in utero [13, 36]. Studies have demonstrated that average height-for-age *z* scores are already low at birth in deprived populations and decline sharply during the first 24 months of life [37]. Our study had similar results. Stunting was lowest in the first 5 months of life (21%) and showed a nearly threefold increase by the time children reached 18–23 months. The steady increase in stunting until 2 years of age highlights the critical period for growth faltering.

A major component of care in the first 23 months is the set of Infant and Young Child Feeding (IYCF) practices recommended by WHO [38]. Our study showed poor IYCF indicators, particularly in terms of dietary diversity and acceptable diet. The high risk of stunting observed in the study may be linked to lack of appropriate food supplementation during the weaning period. An analysis of Demographic Health Survey data suggests that adherence to child feeding practices was associated with less likelihood of stunting or underweight in children [39]. An analysis of data from multiple countries suggested that improved dietary diversity was associated negatively with stunting among children aged 6–23 months [40]. Improved age-appropriate feeding was associated with less likelihood of stunting in our univariable logistic model but was not significant in the adjusted model. This lack of clear association between the achievement of IYCF indicators and stunting may be explained by poor dietary quality at an individual level, particularly in our context. Availability as well as accessibility of nutritious food in urban slums is variable. It is also possible that frequent exposure to intestinal infections associated with poor sanitation offsets the beneficial effects of appropriate feeding practices [41–43]. Another possible argument is that the effects of IYCF are not manifest as concurrent somatic growth or may not manifest until children are older [41].

The literature suggests that unintended pregnancy may adversely affect a child’s health, perhaps contributing to conscious or unconscious neglect and inadequate nutrition, lack of parental bonding and inattention to health care needs [44–48]. These effects influence the process of stunting, probably beginning in the perinatal period and continuing into childhood [49]. Recent studies suggest that children who had been unwanted at the time of conception had an elevated risk of stunting [50, 51]. Our results were consistent with these studies. In the same vein, shorter birth interval is a risk factor for child undernutrition if the mother’s nutrient reserves become depleted, increasing the risk of intrauterine growth retardation and adversely affecting both infant nutrient stores at birth and nutrient delivery via breast milk [52]. A child born within 2 years of the previous child has a 68% higher risk of dying in the neonatal period (days 0–28) and a 99% higher risk of dying in the post-neonatal period (1–12 months), and mothers with short intervals are at higher risk of birth complications [53]. Almost one third of children in India are born after intervals of less than 24 months, risking the survival and undernutrition of both mother and child [54]. Nearly one fifth of children in our study had birth intervals shorter than 24 months and our findings of the positive association of short birth interval with stunting were consistent with other recent studies [14, 55, 56].

Our study revealed that male children had a higher risk of stunting than females. Recent studies have found similar associations between sex and stunting [57–61]. Most studies attribute these sex differences in nutritional status to biological variances in morbidity between boys and girls in early life [62, 63]. A meta-analysis of sixteen Demographic and Health Surveys of ten countries in sub-Saharan Africa found that male children were more likely to become stunted than females, which might suggest that boys are more vulnerable to health inequalities than their female counterparts in the same age groups [63].

Recent studies have identified several maternal risk factors for childhood stunting, such as maternal age and education and poor maternal health seeking behaviour in lower-income countries [56, 60, 64]. An analysis of 180 Demographic and Health Surveys from 62 low- and middle-income countries reported that higher maternal education levels were associated with lower childhood undernutrition [65]. Recent studies in African countries have also suggested that children were significantly less likely to be malnourished when their mothers were more educated [66, 67]. Our findings were in line with these and suggested that higher level of maternal education beyond the tenth grade was a protective factor against stunting. We did not find associations of maternal age, poor antenatal or delivery care with stunting.

Another increasingly evident maternal risk factor is the linkage between high rates of intimate partner violence against women and poor nutritional outcomes in children [68–70]. The poor physical and mental health of mothers who are survivors may affect childcare in many dimensions. Maternal exposure to physical violence substantially increased a child’s risk of stunting in our study.

At the household level, evidence suggests that children in households in the poorest socio-economic groups have higher prevalence of malnutrition [71–73]. This emphasizes the impact of the differential availability of resources to families that act as a major determinant of malnutrition. Household socio-economic position remains a key determinant of nutritional achievement among children [74]. In multivariate analysis, an association between favourable household socio-economic position and better nutritional status was not observed in our study. This is probably explained by the fact that socio-economic differences may be minimal within a sample who all lived in slum conditions and were described as poor. Factors allied to stunting such as inadequate food intake, recurrent illness and poor child care practices are common in slums and affect residents across socio-economic strata.

The strengths of the study were its location in urban slum settlements, its large sample size and the quality of anthropometric data collected. Its main limitation was the cross-sectional nature of the study design, which limited causal inference and documentation of child growth patterns. Residual confounding is also possible. Covariates describing some potential risk factors for malnutrition, such as infection, birth weight and maternal body mass index or height, were not available.

## Conclusion

Our findings support a multidimensional aetiology for stunting. While findings such as the importance of maternal education are consistent with the current literature, the study highlights other protective factors that could be used to influence nutritional outcomes in the shorter term. These include interventions that focus on increased spacing between two pregnancies, preventing unwanted pregnancies and reducing violence experienced by caregivers in the household. The results of the study emphasize the importance of women’s status and decision-making power in urban India, along with access to and uptake of family planning and services to provide support for survivors of domestic violence. Ultimately, a multilateral effort is needed to ensure the success of nutrition-specific interventions by focusing on the underlying health and social status of women living in urban slums.

## Data Availability

All data generated or analysed during this study are available from the corresponding author on reasonable request.

## Abbreviations

WHO: World Health Organization
UNICEF: United Nations Children’s Fund
IYCF: Infant and Young Child Feeding
TEM: Technical error of measurement
HAZ: Height-for-age *z* score
WASH: Water, sanitation and hygiene

## References

[1] UNICEF/WHO/World Bank Group (2017). The World Bank. Joint child malnutrition estimates—Levels and trends (2017 edition).

[2] Achadi E, Ahuja A, Bendech MA, Bhutta ZA, De-Regil LM, Fanzo J, Fracassi P, Grummer-Strawn LM, Haddad LJ, Hawkes CKE (2016). Global nutrition report 2016: from promise to impact: ending malnutrition by 2030.

[3] International Institute for Population Sciences (IIPS) and ICF. National Family Health Survey (NHFS-4), 2015–16: India: International Institute for Population Sciences; 2017.

[4] International Institute for Population Sciences. National Family Health Survey (NFHS-3), 2005-06: India: International Institute for Population Sciences; 2007.

[5] World Health Organization (2017). Global Nutrition Monitoring Framework: operational guidance for tracking progress in meeting targets for 2025.

[6] Office of the Registrar General and Census Commissioner. Government of India; Ministry of Home Affairs: Census of India; 2011.

[7] Ghosh S, Shah D (2004). Nutritional problems in urban slum children. Indian Pediatr.

[8] Savanur MS, Ghugre PS (2015). Magnitude of undernutrition in children aged 2 to 4 years using CIAF and conventional indices in the slums of Mumbai city. J Health Popul Nutr.

[9] Sahoo DP, Dehmubed A, Jajulwar MB (2017). An epidemiological study of acute malnutrition in children of age 6 months to 5 years in an urban slum of Mumbai , Maharashtra. J Datta Meghe Inst Med Sci Univ.

[10] Das S, Bapat U, More NS, Alcock G, Fernandez A, Osrin D (2012). Nutritional status of young children in Mumbai slums: a follow-up anthropometric study. Nutr J.

[11] Mberu BU, Haregu TN, Kyobutungi C, Ezeh AC (2016). Health and health-related indicators in slum, rural, and urban communities: a comparative analysis. Glob Health Action.

[12] World Health Organization. WHO child growth standards : length/height-for-age, weight-for-age, weight-for-length, weight -for-height and body mass index-for-age : methods and development: World Health Organization; 2006.

[13] De Onis M, Branca F, De Onis M, Branca F (2016). Childhood stunting: a global perspective. Matern Child Nutr.

[14] Gribble JN, Murray NJ, Menotti EP (2009). Reconsidering childhood undernutrition: can birth spacing make a difference? An analysis of the 2002-2003 El Salvador National Family Health Survey. Matern Child Nutr.

[15] Stewart CP, Iannotti L, Dewey KG, Michaelsen KF, Onyango AW (2013). Contextualising complementary feeding in a broader framework for stunting prevention. Matern Child Nutr.

[16] Leroy JL, Ruel M, Habicht JPFE (2014). Linear growth deficit continues to accumulate beyond the first 1000 days in low- and middle-income countries: global evidence from 51 national surveys. J Nutr.

[17] Aguayo VM, Nair R, Badgaiyan N, Krishna V (2016). Determinants of stunting and poor linear growth in children under 2 years of age in India: an in-depth analysis of Maharashtra’s comprehensive nutrition survey. Matern Child Nutr.

[18] Kumar A, Singh A (2013). Decomposing the gap in childhood undernutrition between poor and non-poor in urban India , 2005-06. PLoS ONE.

[19] Mullen PM, Nair D, Nigam JSK. Urban health advantages and penalties in India : overview and case studies-discussion paper: The World Bank; 2016.

[20] More NS, Das S, Bapat U, Alcock G, Manjrekar S, Kamble V (2017). Community resource centres to improve the health of women and children in informal settlements in Mumbai: a cluster-randomised, controlled trial. Lancet Glob Health.

[21] Municipal Corporation of Greater Mumbai (2010). Mumbai Human Development Report 2009.

[22] Osrin D, Das S, Bapat U, Alcock GA, Joshi W, More NS (2011). A rapid assessment scorecard to identify informal settlements at higher maternal and child health risk in Mumbai. J Urban Health.

[23] Buckler JM, Green M (2004). A comparison of the early growth of twins and singletons. Ann Hum Biol.

[24] World Health Organization. Global Strategy on Infant and Young Child Feeding (WHA55 A55/15): World Health Organization; 2008. p. 19.

[25] World Health Organization. Physical status: The use of and interpretation of anthropometry, report of a WHO Expert Committee: World Health Organization; 1995. 8594834

[26] World Health Organisation (WHO). Stunted growth and development: context, causes and consequences: World Health Organisation; 2017.

[27] Beal T, Tumilowicz A, Sutrisna A, Izwardy D, Neufeld LM (2018). A review of child stunting determinants in Indonesia. Matern Child Nutr.

[28] Torlesse H, Cronin AA, Sebayang SK, Nandy R (2016). Determinants of stunting in Indonesian children: evidence from a cross-sectional survey indicate a prominent role for the water, sanitation and hygiene sector in stunting reduction. BMC Public Health.

[29] Vyas S, Kumaranayake L, Vyas SKL (2006). Constructing socio-economic status indices: how to use principal components analysis. Health Policy Plan.

[30] World Health Organization. Report of a WHO technical consultation on birth spacing: Geneva, Switzerland 13-15 June 2005: World Health Organization; 2007.

[31] Popat CN, Chaudhari AI, Mazumdar VS, Patel SV (2014). Original Article A cross sectional study to measure the prevalence of malnutrition and factors associated with malnutrition among under five children of an urban slum of Vadodara city. J Res Med Dent Sci.

[32] Sarkar R, Sivarathinaswamy P, Thangaraj B, Sindhu KNC, Ajjampur SSR, Muliyil J (2013). Burden of childhood diseases and malnutrition in a semi-urban slum in southern India. BMC Public Health.

[33] Mittal A, Singh JAS (2007). Effect of maternal factors on nutritional status of 1-5-year-old children in urban slum population. Indian J Community Med.

[34] Cumming O, Cairncross S (2016). Can water, sanitation and hygiene help eliminate stunting? Current evidence and policy implications. Matern Child Nutr.

[35] Ngure FM, Reid BM, Humphrey JH, Mbuya MN, Pelto G, Stoltzfus RJ (2014). Water, sanitation, and hygiene (WASH), environmental enteropathy, nutrition, and early child development: making the links. Ann N Y Acad Sci.

[36] Dewey KG, Begum K (2011). Long-term consequences of stunting in early life. Matern Child Nutr.

[37] de Onis VCG, M. (2010). HPCBM \textbackslash& SR. Worldwide timing of growth faltering: revisiting implications for interventions. Pediatrics..

[38] World Health Organization. Indicators for assessing infant- and young childfeeding practices. Part 1: definitions: conclusions of a consensus meeting held 6-8 November 2007 in Washington DC, USA: World Health Organization; 2008.

[39] Marriott BP, White A, Hadden L, Davies JC, Wallingford JC (2012). World Health Organization (WHO) infant and young child feeding indicators: associations with growth measures in 14 low-income countries: WHO core feeding indicators and growth. Matern Child Nutr.

[40] Ruel MT (2003). Is dietary diversity an indicator of food security or dietary quality? A review of measurement issues and research needs. Food Nutr Bull.

[41] Bentley A, Das S, Alcock G, More NS, Pantvaidya S, Osrin D (2015). Malnutrition and infant and young child feeding in informal settlements in mumbai, india: Findings from a census. Food Sci Nutr.

[42] Baker KK, O’Reilly CE, Levine MM, Kotloff KL, Nataro JP, Ayers TL (2016). Sanitation and hygiene-specific risk factors for moderate-to-severe diarrhea in young children in the Global Enteric Multicenter Study, 2007–2011: case-control study. PLoS Med.

[43] Crocker J, Bartram J (2016). Interpreting the Global Enteric Multicenter Study (GEMS) findings on sanitation, hygiene, and diarrhea. PLoS Med.

[44] Parnell AM, DaVanzo JFW. Contraceptive use and controlled fertility: health issues for women and children: background papers. Committee on Population. Contraceptive use and controlled fertility: health issues for women and children background papers: National Academies Press (US); 1989. 25144029

[45] Guterman K (2015). Unintended pregnancy as a predictor of child maltreatment. Child Abuse Negl.

[46] David HP (2006). Born unwanted, 35 years later: the Prague study. Reprod Health Matters.

[47] Barber JS, Axinn WG, Thornton A (1999). Unwanted childbearing, health, and mother-child relationships. J Health Soc Behav.

[48] Baydar N (1995). Consequences for children of their birth planning status. Fam Plan Perspect.

[49] Shapiro-Mendoza C, Selwyn BJ, Smith DP, Sanderson M (2005). Parental pregnancy intention and early childhood stunting: findings from Bolivia. Int J Epidemiol.

[50] Rahman MM (2015). Is Unwanted birth associated with child malnutrition in Bangladesh?. Int Perspect Sex Reprod Health.

[51] Marston C, Cleland J (2003). Do unintended pregnancies carried to term lead to adverse outcomes for mother and child? An assessment in five developing countries. Popul Stud.

[52] Dewey KG, J. (2007). CR. Does birth spacing affect maternal or child nutritional status? A systematic literature review. Matern Child Nutr.

[53] NIMS I U (2012). Infant and child mortality in India: levels, trends and determinants.

[54] Rutstein SO. Trends in birth spacing DHS comparative reports 28: United States Agency for International Development (USAID); 2011.

[55] Rana MJ, Goli S (2018). Does planning of births affect childhood undernutrition? Evidence from demographic and health surveys of selected South Asian countries. Nutrition..

[56] Das S, Hossain MZ, Islam MA (2008). Predictors of child chronic malnutrition in Bangladesh. Cell..

[57] Chirande L, Charwe D, Mbwana H, Victor R, Kimboka S, Issaka AI (2015). Determinants of stunting and severe stunting among under-fives in Tanzania: evidence from the 2010 cross-sectional household survey. BMC Pediatr.

[58] Bork KA, Diallo A (2017). Boys are more stunted than girls from early infancy to 3 years of age in rural Senegal. J Nutr.

[59] Jawaregowda S, Angadi M (2015). Gender differences in nutritional status among under five children in rural areas of Bijapur district, Karnataka, India. Int J Commun Med Public Health.

[60] Adekanmbi VT, Kayode GA, Uthman OA (2013). Individual and contextual factors associated with childhood stunting in Nigeria: a multilevel analysis. Matern Child Nutr.

[61] Baschieri A, Machiyama K, Floyd S, Dube A, Molesworth A, Chihana M (2017). Unintended childbearing and child growth in Northern Malawi. Matern Child Health J.

[62] Garenne M (2003). Sex differences in health indicators among children in African DHS surveys. J Biosoc Sci.

[63] Wamani H, Åstrøm AN, Peterson S, Tumwine JK, Tylleskär T (2007). Boys are more stunted than girls in Sub-Saharan Africa: a meta-analysis of 16 demographic and health surveys. BMC Pediatr.

[64] Siddiqi MN, Haque MNGM (2011). Malnutrition of under-five children: evidence from Bangladesh. Asian J Med Sci.

[65] Vollmer S, Bommer C, Krishna A, Harttgen K, Subramanian SV (2017). The association of parental education with childhood undernutrition in low- and middle-income countries: comparing the role of paternal and maternal education. Int J Epidemiol.

[66] Makoka D, Masibo PK (2015). Is there a threshold level of maternal education sufficient to reduce child undernutrition? Evidence from Malawi, Tanzania and Zimbabwe. BMC Pediatr.

[67] Hamel C, Enne J, Omer K, Ayara N, Yarima Y, Cockcroft A (2015). Childhood malnutrition is associated with maternal care during pregnancy and childbirth: a cross-sectional study in Bauchi and cross river states, Nigeria. J Public Health Res.

[68] Rahman M, Poudel KC, Yasuoka J, Otsuka K, Yoshikawa K (2012). Maternal exposure to intimate partner violence and the risk of undernutrition among children younger than 5 years in Bangladesh. Am J Public Health.

[69] Chai J, Fink G, Kaaya S, Danaei G, Fawzi W, Ezzati M (2016). Association between intimate partner violence and poor child growth : results from 42 demographic and health surveys. Bull World Health Organ.

[70] Ziaei S, Naved RT, Ekström EC (2014). Women’s exposure to intimate partner violence and child malnutrition: findings from demographic and health surveys in Bangladesh. Matern Child Nutr.

[71] Rabbani A, Khan A, Yusuf S, Adams A (2016). Trends and determinants of inequities in childhood stunting in Bangladesh from 1996/7 to 2014. Int J Equity Health.

[72] Islam MR, Rahman MS, Rahman MM, Nomura S, de Silva A, Lanerolle P (2020). Reducing childhood malnutrition in Bangladesh: the importance of addressing socio-economic inequalities. Public Health Nutr.

[73] Husseini M, Darboe MK, Moore SE, Nabwera HM, Prentice AM (2018). Thresholds of socio-economic and environmental conditions necessary to escape from childhood malnutrition: a natural experiment in rural Gambia. BMC Med.

[74] Kanjilal B, Mazumdar PG, Mukherjee M, Rahman MH (2010). Nutritional status of children in India: household socio-economic condition as the contextual determinant. Int J Equity Health.

